# Integrative analysis of Helicobacter pylori-driven stomach adenocarcinoma reveals epigenetic deregulation, immune evasion, and therapeutic resistance

**DOI:** 10.1186/s41065-025-00616-z

**Published:** 2025-11-25

**Authors:** Mohammed Alissa

**Affiliations:** Department of Medical Laboratory, College of Applied Medical Sciences, Prince Sattam bin Abdulaziz University, Al-Kharj, 11942 Saudi Arabia

**Keywords:** STAD, Helicobacter pylori (H. pylori), Carcinogenesis, Diagnosis, Prognosis

## Abstract

**Background:**

Helicobacter pylori (H. pylori) infection is a major etiological factor for stomach adenocarcinoma (STAD), yet the key molecular drivers linking infection to tumor progression remain insufficiently defined. This study aimed to identify H. pylori–related hub genes in STAD and validate their functional relevance using integrated bioinformatics and experimental approaches.

**Methodology:**

Differentially expressed genes (DEGs) were identified from two microarray datasets (GSE13911 and GSE54129) comparing H. pylori-positive STAD samples with controls. Common DEGs were used to construct a protein–protein interaction (PPI) network via STRING and Cytoscape, and hub genes were ranked using CytoHubba. Transcriptomic validation was conducted using TCGA-STAD data, followed by analyses of enrichment pathways, promoter methylation, somatic mutations, CNVs, immune subtype associations, and drug sensitivity using GSCA, UALCAN, cBioPortal, and CTRP datasets. miRNA–mRNA regulatory interactions were predicted using miRNet and validated in vitro. Experimental validation included RT-qPCR, Western blotting, CCK-8 proliferation assays, colony formation, and wound-healing assays in MKN45 and AGS cells following siRNA-mediated knockdown of key hub genes. Additionally, AGS cells were infected with live H. pylori to directly assess infection-induced changes in gene expression and malignant phenotypes.

**Results:**

Four hub genes (THBS2, CTNNB1, COL4A1, and E2F3) were identified as commonly upregulated in H. pylori-positive STAD samples and were further validated as highly expressed in STAD tissues and cell lines. Promoter hypomethylation and CNV gains contributed to their overexpression. Pathway analyses linked the hub genes to EMT, cell cycle progression, immune suppression, and oncogenic signaling. miRNA profiling identified hsa-miR-9-3p and hsa-miR-9-5p as common regulators with diagnostic potential. Importantly, H. pylori infection of AGS cells induced strong upregulation of COL4A1 and CTNNB1 and significantly increased proliferation, clonogenicity, and migration, demonstrating a direct infection-driven oncogenic response. Conversely, siRNA-mediated silencing of COL4A1 or CTNNB1 markedly reduced proliferation, colony formation, and wound closure, confirming their functional roles in STAD progression. Immune correlation and drug sensitivity analyses further linked high hub-gene expression to immunosuppressive microenvironments and resistance to multiple therapeutic agents.

**Conclusion:**

This study identifies THBS2, CTNNB1, COL4A1, and E2F3 as key H. pylori-associated oncogenic drivers in STAD. Functional assays demonstrate that H. pylori enhance malignant phenotypes through COL4A1 and CTNNB1, while gene silencing reverses these effects. These findings highlight the hub genes and their regulatory miRNAs as promising diagnostic biomarkers and potential therapeutic targets in H. pylori-related gastric cancer.

**Supplementary Information:**

The online version contains supplementary material available at 10.1186/s41065-025-00616-z.

## Introduction

Stomach adenocarcinoma (STAD) remains a major global health burden, ranking among the top causes of cancer related mortality, particularly in East Asia, Eastern Europe, and parts of South America and Central America [1–3]. According to GLOBOCAN 2024 estimates, over one million new STAD cases and approximately seven hundred seventy thousand related deaths occurred worldwide, highlighting the urgent need for improved prevention and treatment strategies [2, 4, 5]. Despite notable advancements in surgical techniques, chemoradiotherapy, and targeted molecular therapies including HER2 inhibitors and immune checkpoint blockade, the prognosis for advanced stage STAD remains dismal, with a five-year survival rate typically below 30% [6–8]. Sonkin et al. have shown that the historical progression of cancer treatment has moved from broadly cytotoxic regimens toward highly specific molecular and immune mediated approaches, yet they also emphasise that only a minority of patients with solid tumours experience substantial clinical benefit from current immunotherapies, which highlights the need for new mechanistic insights and improved therapeutic targets in cancers such as STAD [9–11].

Among the established etiological factors, chronic infection with Helicobacter pylori (H. pylori), a Gram negative, spiral shaped, microaerophilic bacterium, has been recognised as a predominant and modifiable risk factor for gastric carcinogenesis. H. pylori infect more than half of the global population, with infection rates exceeding 70% in some developing countries [12, 13]. Upon colonisation of the gastric mucosa, H. pylori induce a persistent inflammatory response and mucosal damage through the secretion of various virulence factors, notably Cytotoxin associated gene A (CagA) and Vacuolating cytotoxin A (VacA) [14–16]. These proteins interfere with host cell signalling, disrupt epithelial cell junctions, promote DNA damage, and modulate immune responses, ultimately leading to a cascade of events known as the Correa cascade, which includes chronic gastritis, atrophic gastritis, intestinal metaplasia, dysplasia, and eventual malignant transformation [14, 15]. Importantly, strains carrying the cag pathogenicity island, which encodes a type IV secretion system responsible for translocating CagA into gastric epithelial cells, are significantly associated with increased cancer risk. CagA positive strains activate oncogenic pathways such as NF kappa B, ERK MAPK, and PI3K AKT, driving cellular proliferation, anti apoptotic signalling, and immune evasion [17, 18]. VacA, on the other hand, induces mitochondrial dysfunction and epithelial cell vacuolation, further contributing to tissue injury and immune modulation. In recognition of its carcinogenic potential, the World Health Organization and the International Agency for Research on Cancer classified H. pylori as a class I carcinogen in 1994 [19].

Accumulating studies have demonstrated that H. pylori infection alters host gene expression and signalling pathways that are critical for inflammation, epithelial mesenchymal transition, immune evasion, and cell proliferation. For example, Pachathundikandi et al. reported that H. pylori infection induces the upregulation of IL1B, CXCL8, and PTGS2, contributing to an inflammatory microenvironment conducive to tumorigenesis [20]. Another study by Chaithongyot et al. identified MMP9, TLR2, and NFKBIA as H. pylori responsive genes that mediate immune dysregulation in gastric epithelial cells [21]. Similarly, Lim et al. revealed that CCL20, BIRC3, and TNFAIP3 were significantly upregulated in H. pylori infected gastric tissues, implicating these genes in activation of NF kappa B and anti apoptotic pathways [22]. Beyond these gene specific findings, transcriptomic studies have provided deeper insights into network level dysregulation. For instance, Bao et al. utilised weighted gene co expression network analysis to identify CXCL1, CD44, and S100A9 as central hub genes in H. pylori positive STAD samples [23]. Importantly, another study demonstrated that H. pylori infection directly enhances the migration of gastric cancer cells and is associated with poorer clinical outcomes. This work provided functional evidence that H. pylori promote cytoskeletal reorganisation, increased motility, and early metastatic behaviour [24]. Earlier investigations have also demonstrated that H. pylori mediated activation of the Wnt beta catenin pathway contributes to malignant transformation, underscoring the importance of CTNNB1 in gastric carcinogenesis [25]. Concurrently, substantial evidence indicates that extracellular matrix remodelling genes such as COL4A1 play critical roles in gastric cancer invasion and progression, linking matrix alterations with H. pylori induced oncogenic processes [26]. An important related study titled H. pylori infection facilitates cell migration and potentially impacts clinical outcomes in gastric cancer specifically showed how H. pylori enhance motility and promotes clinically relevant phenotypic changes [27]. The lack of integration of this evidence into many transcriptomic studies has limited the interpretation of functional significance. By incorporating this prior work, the rationale of the present study becomes clearer and more grounded in recent experimental literature. In addition, recent computational advances such as generative adversarial networks applied to gene expression analysis have provided novel tools for modelling transcriptomic variation. These approaches highlight the importance of selecting robust datasets and appropriate feature selection strategies when identifying hub genes. However, the landscape of H. pylori related hub genes in STAD remains incompletely defined, and further exploration is warranted to identify novel diagnostic markers and therapeutic targets. Based on the convergence of prior evidence involving Wnt beta catenin signalling and extracellular matrix remodelling, we selected CTNNB1 and COL4A1 as biologically plausible candidates for further investigation.

In this study, we aimed to identify and characterise H. pylori associated hub genes in STAD using a comprehensive in silico [28, 29] and in vitro approach [30, 31]. By integrating previous transcriptomic evidence with functional experimentation, and by situating our objectives within the context of recent findings on H. pylori mediated cell migration and signaling, the present study seeks to fill an important gap by linking hub gene prediction with experimentally validated biological relevance. Our integrative analysis provides new insights into the molecular mechanisms of H. pylori mediated STAD carcinogenesis and proposes novel gene targets for future clinical investigation.

## Methodology

### Cell lines and culture conditions

A total of eight human STAD cell lines, including MKN45, AGS, SNU-1, SNU-5, NCI-N87, HGC-27, KATO III, and MKN28 and five normal human gastric epithelial cell lines, including GES-1, HFE-145, GES-1 C, GES-1 A, and Hs738.St/Int were purchased from commercial cell repositories including ATCC (American Type Culture Collection, USA) and Procell Life Science & Technology Co., Ltd. (Wuhan, China). All cell lines were authenticated via short tandem repeat (STR) profiling and tested negative for Mycoplasma contamination. Cells were cultured under standard conditions in RPMI-1640 medium (Thermo Fisher Scientific, USA) supplemented with 10% fetal bovine serum (FBS; Gibco, USA) and 1% penicillin–streptomycin at 37 °C in a humidified atmosphere containing 5% CO₂. Medium was replaced every 2–3 days, and cells were subcultured upon reaching 70–80% confluency using 0.25% trypsin-EDTA (Gibco).

### RNA extraction and real-time quantitative PCR (RT-qPCR)

Total RNA was extracted from cultured cell lines using the TRIzol™ reagent (Thermo Fisher Scientific, USA) according to the manufacturer’s protocol [32]. For cDNA synthesis, 1 µg of total RNA was reverse transcribed using the High-Capacity cDNA Reverse Transcription Kit (Applied Biosystems, USA) following the recommended protocol [33]. RT-qPCR was carried out using PowerUp™ SYBR™ Green Master Mix (Applied Biosystems, USA) on a QuantStudio™ 5 Real-Time PCR System (Applied Biosystems) [34]. Gene-specific primers for THBS2, CTNNB1, COL4A1, and E2F3 were purchased commercially. GAPDH was used as the internal control. Relative gene expression was calculated using the 2^^−ΔΔCt^ method [35]. Following primers were used for amplification purpose.

GAPDH-F 5’-ACCCACTCCTCCACCTTTGAC-3’,

GAPDH-R 5’-CTGTTGCTGTAGCCAAATTCG-3’

THBS2-F:5’-CAGTCTGAGCAAGTGTGACACC-3’

THBS2-R: 5’-TTGCAGAGACGGATGCGTGTGA-3’

CTNNB1-F: 5’-CACAAGCAGAGTGCTGAAGGTG − 3’

CTNNB1-R: 5’-GATTCCTGAGAGTCCAAAGACAG-3’

COL4A1-F: 5’-TGTTGACGGCTTACCTGGAGAC-3’

COL4A1-R: 5’-GGTAGACCAACTCCAGGCTCTC-3’

E2F3-F: 5’-AGCGGTCATCAGTACCTCTCAG-3’

E2F3-R: 5’-TGGTGAGCAGACCAAGAGACGT-3’

### Microarray dataset selection and preprocessing

To identify key genes associated with H. pylori infection in STAD, two publicly available gene expression datasets, GSE13911 and GSE54129, were retrieved from the Gene Expression Omnibus (GEO) database (https://www.ncbi.nlm.nih.gov/geo/) [36]. Both datasets were generated on the Affymetrix Human Genome U133 Plus 2.0 Array platform (GPL570). Raw expression data were downloaded and pre-processed using the R programming environment (version 4.3.0). Background correction, normalization, and log2 transformation were performed using the “affy” and “limma” packages in Bioconductor. Probes were mapped to gene symbols, and in cases of multiple probes mapping to a single gene, the probe with the highest average expression was selected. The datasets were selected because both contain well characterised H. pylori infected and non infected gastric tissue samples, consistent with earlier microarray-based investigations by Roy et al. (GSE13861) [37] and Mohamed et al. (GSE27411) [38], which demonstrated that robust H. pylori associated transcriptional patterns require adequate sample representation and careful preprocessing. The inclusion of two independent cohorts also follows recommendations from previous gastric infection transcriptomic studies emphasising cross validation to minimise dataset specific artefacts [39, 40].

### Differential expression analysis

Differentially expressed genes (DEGs) between H. pylori-positive tumor tissues and normal controls were identified independently for each dataset using the “limma” package. Genes with adjusted P-values < 0.05 and absolute log2 fold change (|log₂FC|) >1 were considered significantly dysregulated. The top 2,000 DEGs from each dataset were selected for further comparison. Overlapping DEGs common to both datasets were identified using the VennDiagram R package to ensure consistency and robustness of gene expression alterations associated with H. pylori infection. This consensus-based approach is supported by prior multi cohort analyses such as those by Mohamed et al. [38] and Li et al. [41], which showed that overlapping DEGs across independent H. pylori datasets provide more reliable infection associated signatures than single dataset analyses.

### Hub gene identification

The commonly dysregulated genes were submitted to the STRING database (Search Tool for the Retrieval of Interacting Genes/Proteins, version 11.5; https://string-db.org) [42] to construct a high-confidence PPI network. An interaction score threshold of ≥ 0.7 (high confidence) was applied to reduce spurious interactions. The resulting network was visualized and analyzed using Cytoscape software (version 3.9.1) [43]. To identify central hub genes within the PPI network, topological analysis was performed using the CytoHubba plugin in Cytoscape. The degree algorithm, which quantifies the number of direct connections for each node, was applied to prioritize the most interconnected and potentially biologically significant genes. The top-ranking hub genes were selected for downstream validation and functional analysis. This workflow reflects the methodological framework used in previous H. pylori related systems biology studies, including Mohammed et al. [38] and Baj et al. [44], which identified CXCL1, CD44, and S100A9 as infection associated regulatory hubs. Given strong evidence that H. pylori activate Wnt beta catenin signalling and promotes extracellular matrix remodelling through collagen related pathways [45, 46], CTNNB1 and COL4A1 were prioritised during downstream analysis as biologically relevant hub candidates.

### Transcriptomic validation analysis of hub genes in the cancer genome atlas (TCGA)-STAD cohort

To validate the expression profiles and clinical significance of the identified hub genes in a large cohort, RNA-sequencing and clinical data for STAD were obtained from TCGA via the GSCA platform (http://bioinfo.life.hust.edu.cn/GSCA/) [47]. Gene expression levels were normalized to fragments per kilobase of transcript per million mapped reads (FPKM), and differential expression between tumor and normal tissues was evaluated using the Wilcoxon rank-sum test. Moreover, GSCA database was also used for the gene set enrichment analysis (GSEA) of the hub genes in STAD.

### Survival analysis

The prognostic relevance of hub genes was evaluated in STAD patients using Kaplan–Meier survival analysis within the GSCA web server [47].

### Immunohistochemical validation of hub gene expression in STAD tissues

To confirm the protein-level expression of the identified hub genes in gastric cancer tissues, immunohistochemistry (IHC) staining data were retrieved from the HPA database (https://www.proteinatlas.org/) [48]. IHC staining patterns, intensity, and localization for each protein were evaluated in gastric cancer tissues and compared to normal gastric mucosa samples.

### Analysis of promoter methylation and pathway activity of hub genes

To investigate the epigenetic regulation of the hub genes promoter methylation data were obtained from the UALCAN database (http://ualcan.path.uab.edu/) [49], which provides TCGA-based DNA methylation profiles. Beta values representing methylation levels at promoter regions in normal versus tumor gastric cancer were extracted and statistically compared to evaluate differential methylation patterns. To assess the regulatory relationship between promoter methylation and gene expression, Spearman correlation analysis was conducted using the GSCA platform [47], which integrates multi-omics data from TCGA. Correlation coefficients and false discovery rates (FDR) were computed to determine the significance of inverse methylation–expression relationships. To explore the involvement of hub genes in oncogenic signalling pathways, pathway activity scores were also retrieved from the GSCA database.

### Genetic alteration analysis of hub genes

To explore the genomic alterations in the identified hub genes in STAD, mutational and CNV analyses were conducted using the cBioPortal (https://www.cbioportal.org/) [50].

### Prediction and validation of miRNA–mRNA regulatory interactions

To investigate the post-transcriptional regulatory mechanisms involving the identified hub genes, a miRNA–mRNA interaction network was constructed using the miRNet database (https://www.mirnet.ca/), which integrates data from multiple established miRNA-target prediction algorithms, including miRTarBase, TargetScan, and miRanda [51]. The analysis was conducted by inputting the four hub genes to retrieve candidate miRNAs that potentially target them. The resulting network was visualized and exported for further analysis. To evaluate the expression levels of key predicted miRNAs, the UALCAN database [49] was used to extract miRNA expression profiles from the TCGA-STAD dataset. Additionally, the Kaplan–Meier Plotter module of the UALCAN database was used to assess the prognostic significance of the miRNAs. For miRNA quantification, TaqMan™ Advanced miRNA Assays (Thermo Fisher Scientific) were used to detect and quantify hsa-miR-9-3p and hsa-miR-9-5p.

### Immune subtype analysis, immune landscape characterization, and drug sensitivity profiling

To evaluate the immune context of the identified hub genes in STAD, immune subtype-specific gene expression analysis was performed using the TISIDB database (http://cis.hku.hk/TISIDB/) [52]. To further investigate the interaction between hub genes and the immune microenvironment, their correlation with immune modulators was assessed. Correlation analysis between gene expression and immune inhibitory and stimulatory molecules was conducted using Spearman’s rank correlation coefficients in the TISIDB platform. Immune infiltration profiles were assessed using the GSCA database [47]. Drug sensitivity profiling was conducted by correlating hub gene expression with IC_50_ values derived from the CTRP. The GSCA platform was employed to identify associations between gene expression levels and drug response in STAD cell lines.

### Gene enrichment analysis

To investigate the functional landscape and biological relevance of the identified hub genes, a PPI networks were constructed using the Pathway Commons database (https://www.pathwaycommons.org/) [53], which aggregates curated interaction data from multiple sources. For functional annotation, gene enrichment analysis of the hub genes and their PPI interactors was performed using the DAVID v6.8 (https://david.ncifcrf.gov/) [54].

### siRNA-mediated knockdown and Western blot analysis of COL4A1 and CTNNB1

To evaluate the functional role of COL4A1 and CTNNB1, MKN45 and AGS cells were transfected with gene-specific siRNAs (Thermo Fisher Scientific, USA) using Lipofectamine™ RNAiMAX Transfection Reagent (Thermo Fisher Scientific) following the manufacturer’s instructions. A non-targeting siRNA served as the control. After 48 h of transfection, gene silencing efficiency was confirmed at both mRNA and protein levels. respectively. Western blot analysis of the COL4A1 and CTNNB1 was performed according to the protocols published by recent studies [55, 56].

### H. pylori culture

H. pylori (ATCC 43504, CagA⁺/VacA⁺) was cultured on Columbia blood agar supplemented with 5% defibrinated sheep blood (Thermo Fisher Scientific) and incubated under microaerophilic conditions consisting of 5% O₂, 10% CO₂, and 85% N₂ at 37 °C for 48 to 72 h. Bacteria were harvested in Brucella broth (Thermo Fisher Scientific), and the optical density at 600 nm was adjusted to achieve the required multiplicity of infection for downstream experiments.

### H. pylori infection

For infection, AGS cells were seeded in 6 well tissue culture plates (Corning) at a density of 2 × 10⁵ cells per well in 2 mL of complete RPMI 1640 medium and allowed to adhere overnight until they reached approximately 70% to 80% confluence. Twenty-four hours before infection, the medium was replaced with 2 mL of antibiotic free RPMI 1640 containing 10% fetal bovine serum, and cells were washed 2 times with antibiotic free phosphate buffered saline (Gibco, Thermo Fisher Scientific) to remove residual antibiotics. On the day of infection, H. pylori were resuspended in antibiotic free RPMI 1640 with 10% fetal bovine serum, and 2 mL of this bacterial suspension was added per well to achieve a multiplicity of infection of 50 bacteria per AGS cells. Plates were gently rocked by hand to distribute bacteria evenly over the monolayer and then incubated at 37 °C in 5% carbon dioxide for 6, 12, or 24 h according to the experimental design. Uninfected control wells received 2 mL of antibiotic free RPMI 1640 with 10% fetal bovine serum without bacteria and were incubated under the same conditions. At the end of the infection period, cells were washed 3 times with phosphate buffered saline to remove non adherent bacteria before being processed for downstream assays.

### Functional assays

Cell proliferation was assessed using the Cell Counting Kit-8 (Thermo Fisher Scientific). Transfected cells were seeded in 96-well plates and absorbance was measured at 450 nm at indicated time points. Colony formation assays were performed by seeding transfected cells in 6-well plates and allowing colonies to form for 10–14 days, followed by fixation in methanol, staining with 0.1% crystal violet, and manual counting. Wound healing assays were conducted to evaluate migratory potential. Briefly, confluent monolayers of transfected cells were scratched with a pipette tip, washed with PBS, and cultured in serum-free medium. Wound closure was imaged at 0 h and 24 h using an inverted microscope and quantified using ImageJ software.

### Statistical analysis

All experiments were performed in triplicate. Quantitative data are presented as mean ± standard deviation (SD). Statistical comparisons between two groups were conducted using the unpaired two-tailed Student’s t-test, while comparisons among multiple groups were analyzed using one-way ANOVA followed by Tukey’s post hoc test. Correlation analyses were performed using Spearman’s rank correlation coefficient. P-value < 0.05 < 0.01, and < 0.001 were considered statistically significant. All statistical analyses were conducted using GraphPad Prism (version 9.0) and R software (version 4.3.0).

## Results

### Dataset retrieval, identification and validation of hub genes in H. Pylori infection-related STAD samples

**Fig. 1:**
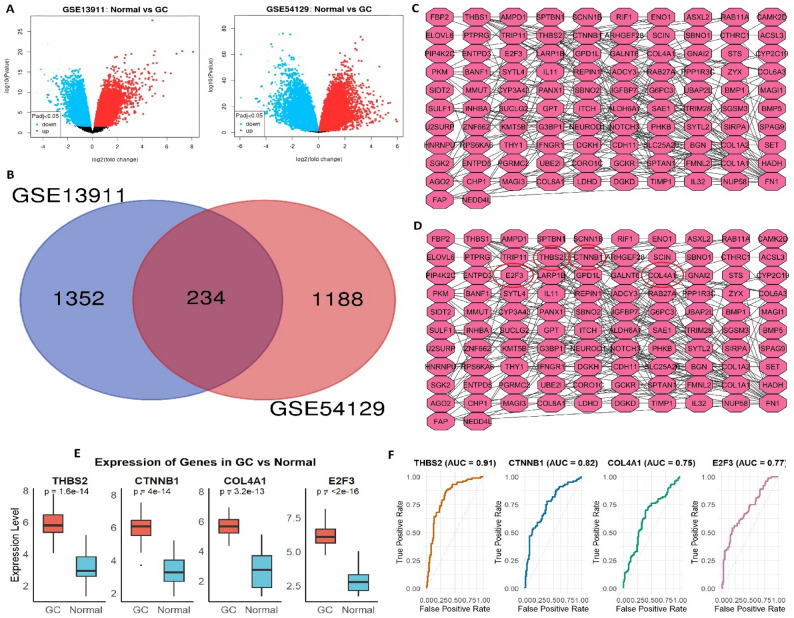
Identification and validation of hub genes in H. pylori-associated stomach adenocarcinoma (STAD). **A** Differentially expressed genes (DEGs) identified from GSE13911 and GSE54129 datasets. **B** Venn diagram showing 234 overlapping DEGs shared between the two datasets. **C** Protein–protein interaction (PPI) network constructed from common DEGs using the STRING database. **D** Top hub genes ranked by degree centrality using the CytoHubba plugin in Cytoscape. **E** RT-qPCR validation of hub gene expression in eight gastric cancer cell lines versus five normal gastric epithelial cell lines. **F** Receiver operating characteristic (ROC) curve analysis showing diagnostic performance (AUC values) of THBS2, CTNNB1, COL4A1, and E2F3 in distinguishing cancerous from normal tissues. P-value < 0.05

Differential gene expression analysis was conducted on two independent H. Pylori infection-related STAD (GSE13911 and GSE54129) obtained from the GEO database. Using the limma package, significantly upregulated and downregulated genes were identified based on adjusted *P* < 0.05 and |log₂ fold change| >1 criteria. A substantial number of DEGs were found in both datasets, indicating robust transcriptional dysregulation associated with H. pylori infection (Fig. 1 A). To determine the shared molecular features between datasets, the top 2000 DEGs from each dataset were compared. A total of 234 genes were commonly dysregulated across both datasets, suggesting conserved expression changes in H. pylori-related STAD (Fig. 1B). These overlapping genes were subjected to PPI network construction using the STRING database, followed by network analysis in Cytoscape. Hub genes were prioritized using the degree method in the CytoHubba plugin, resulting in the identification of key regulatory candidates including THBS2, CTNNB1, COL4A1, and E2F3 as top-ranking nodes within the interaction network (Fig. 1 C and D). Expression levels of the hub genes were next validated by RT-qPCR in eight STAD cell lines and five normal gastric epithelial cell lines. All four genes exhibited significantly elevated expression in STAD cells relative to normal controls, supporting their potential role in H. pylori-mediated tumorigenesis (Fig. 1E). To assess the diagnostic potential of these hub genes, ROC curve analysis was performed. THBS2 showed the highest diagnostic accuracy with an AUC of 0.91, followed by CTNNB1 (AUC = 0.82), E2F3 (AUC = 0.77), and COL4A1 (AUC = 0.75), indicating their utility as potential biomarkers for distinguishing STAD from normal tissue (Fig. 1 F).

### Validation of hub gene expression and clinical relevance in STAD

**Fig. 2:**
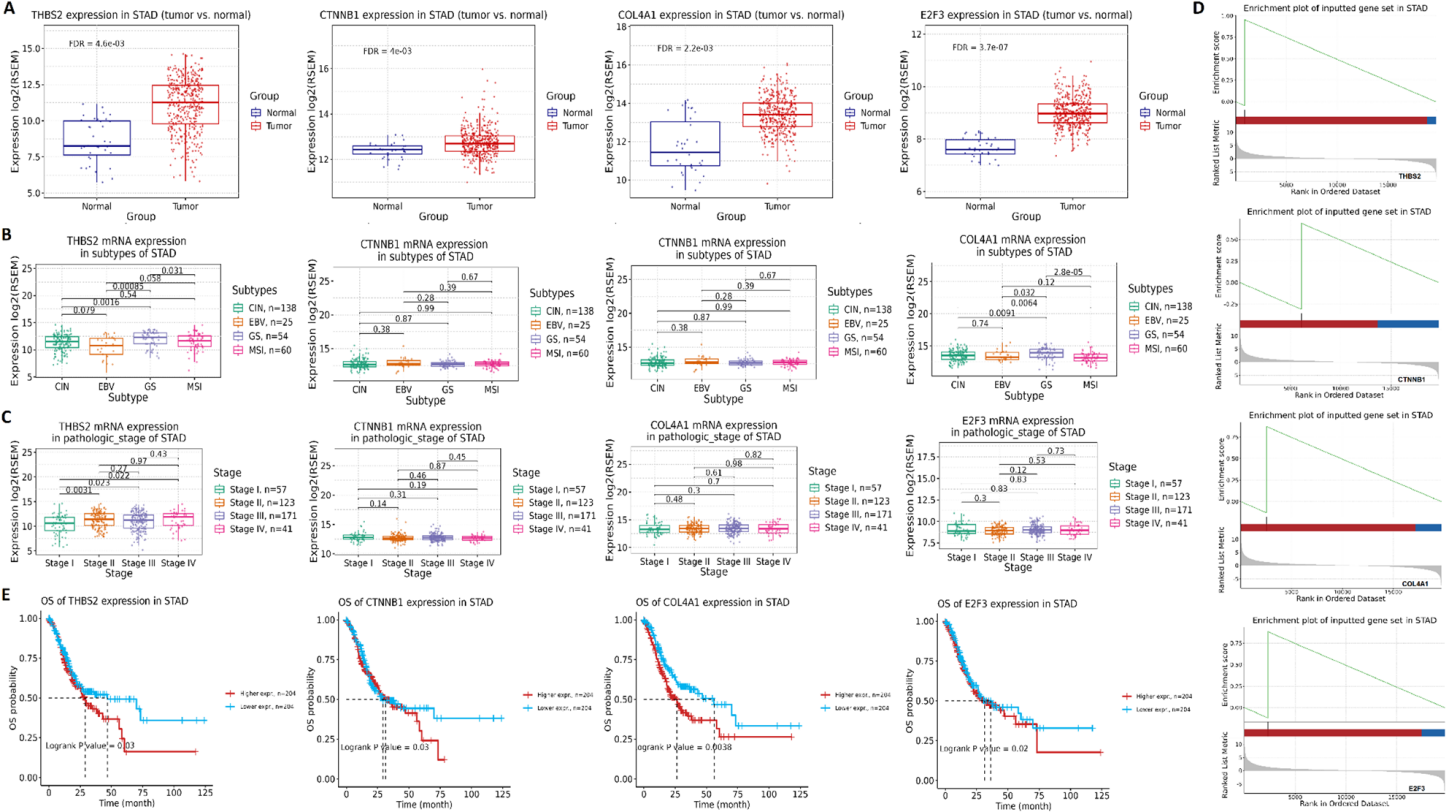
Clinical validation and prognostic relevance of hub genes in STAD using TCGA data. **A** mRNA expression levels of THBS2, CTNNB1, COL4A1, and E2F3 in STAD tumor vs. normal tissues from the TCGA-STAD cohort. **B** Expression of hub genes across molecular subtypes of STAD (CIN, EBV, GS, and MSI). **C** Gene expression patterns stratified by pathological stage. **D** Kaplan–Meier survival analysis indicating that higher expression of all four genes is associated with worse overall survival. **E** Gene set enrichment analysis (GSEA) confirming significant association of hub genes with STAD development. P-value < 0.05

To further validate the expression and clinical significance of the identified hub genes in STAD, we analyzed their mRNA expression, association with molecular subtypes, clinical stages, and patient survival using the TCGA-STAD dataset through the GSCA. The mRNA expression analysis revealed that THBS2, CTNNB1, COL4A1, and E2F3 were significantly upregulated in tumor tissues compared to normal gastric tissues in the TCGA-STAD cohort (Fig. 2 A). Further analysis across molecular subtypes of STAD demonstrated subtype-specific expression differences. THBS2 and COL4A1 were highly expressed in the CIN and EBV subtypes, with significantly lower expression in GS and MSI subtypes (Fig. 2B). CTNNB1 and E2F3 showed moderate but non-significant variations across subtypes, although expression remained elevated in CIN and EBV subgroups (Fig. 2B). Evaluation of gene expression across pathological stages of STAD revealed an increasing trend of COL4A1 expression with disease progression, reaching statistical significance between stage I and stage IV (*P* = 0.048), whereas THBS2, CTNNB1, and E2F3 showed no significant stage-dependent variation (Fig. 2 C). Survival analysis indicated that higher expression of THBS2, CTNNB1, COL4A1 and E2F3 was significantly associated with worse overall survival in STAD (Fig. 2D). GSEA analysis revealed that hub genes were significantly associated with STAD development (Fig. 2E).

### Validation of hub gene expression at the protein level in STAD

**Fig. 3:**
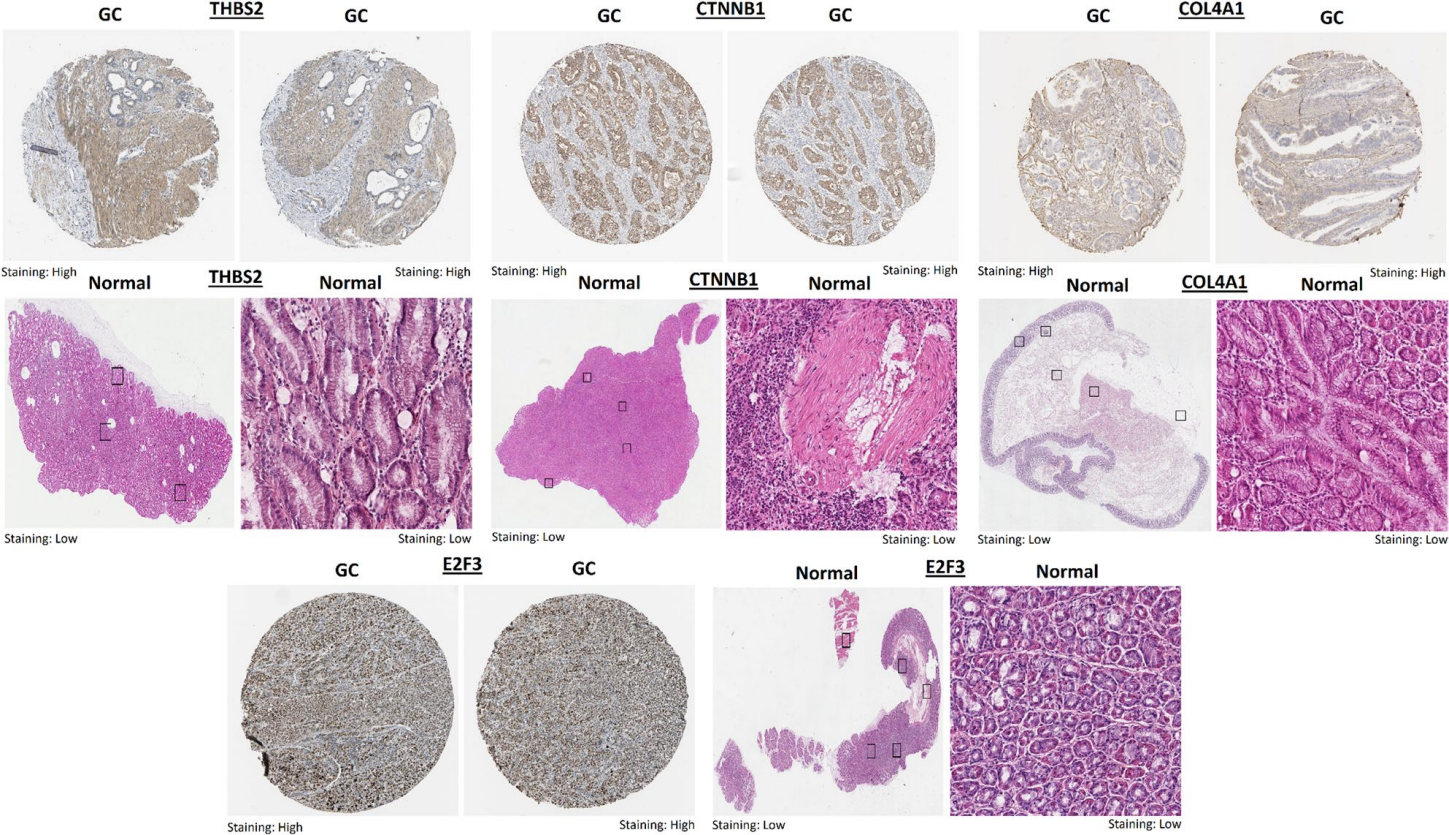
Protein-level validation of hub gene expression in gastric cancer tissues using HPA data. Immunohistochemistry (IHC) analysis from the HPA showing increased protein expression of THBS2, CTNNB1, COL4A1, and E2F3 in gastric cancer tissues compared to normal gastric mucosa

Protein-level validation using immunohistochemistry (IHC) data from the HPA database revealed that THBS2, CTNNB1, COL4A1, and E2F3 exhibited markedly higher staining intensity in gastric cancer tissues compared to normal gastric mucosa (Fig. 3). Specifically, high protein expression of THBS2 was observed in tumor tissues, whereas normal tissues displayed low levels (Fig. 3). Similarly, CTNNB1 showed strong cytoplasmic and membranous staining in cancerous tissues, with significantly weaker expression in corresponding normal samples (Fig. 3). COL4A1 also demonstrated elevated expression in gastric cancer tissues, while normal samples showed minimal staining (Fig. 3). E2F3 staining was strongly positive in gastric cancer sample sections but was weak or absent in normal tissues (Fig. 3).

### Epigenetic regulation and pathway activity of hub genes in STAD

**Fig. 4:**
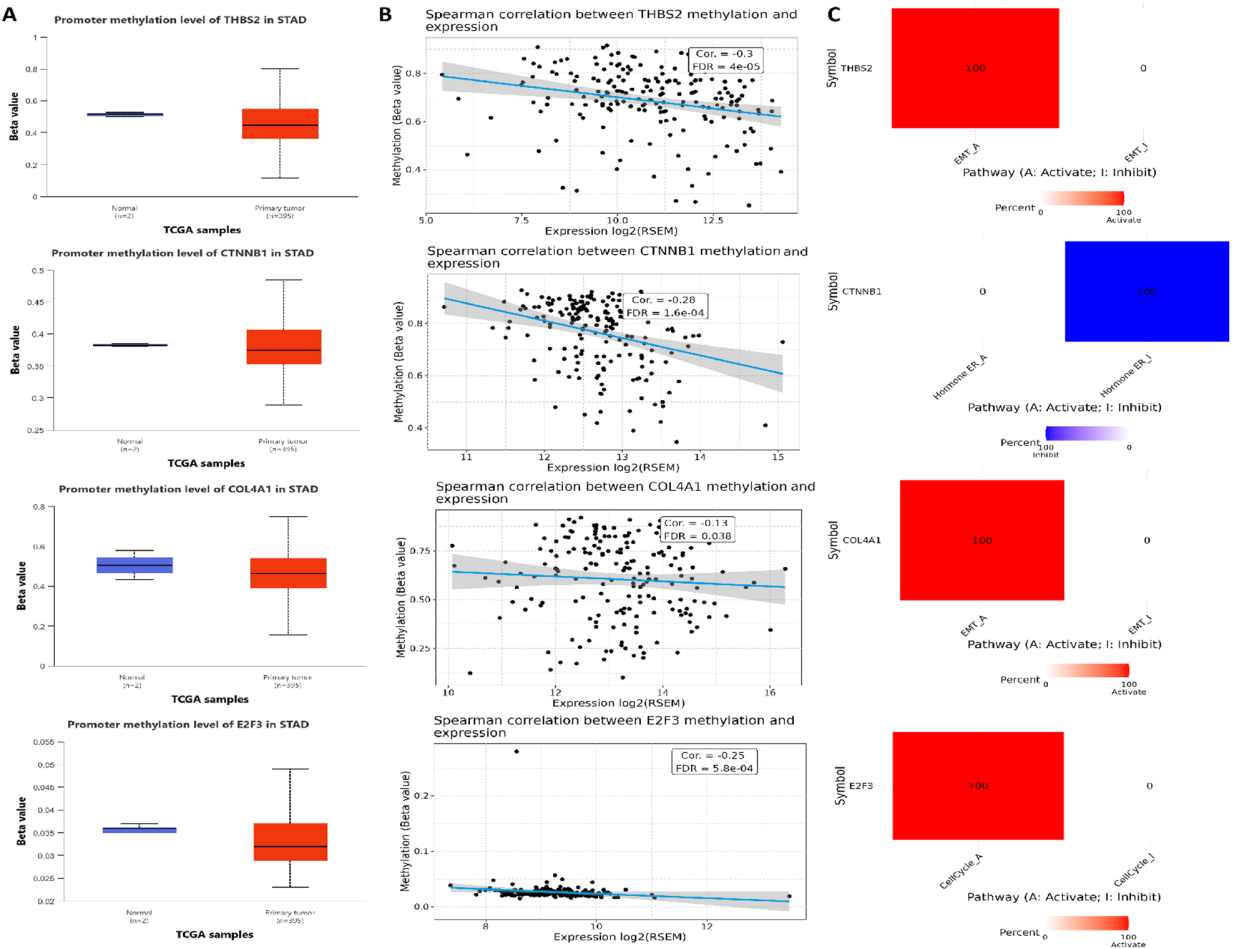
Epigenetic regulation and pathway activity of hub genes in STAD. **A** Promoter methylation levels of THBS2, CTNNB1, COL4A1, and E2F3 in tumor vs. normal tissues using UALCAN database. **B** Correlation analysis between promoter methylation and gene expression using GSCA database. **C** Pathway activity profiles showing functional enrichment: THBS2, COL4A1, and E2F3 activate epithelial–mesenchymal transition (EMT) pathways; CTNNB1 is associated with suppression of hormone receptor pathways; E2F3 additionally activates cell cycle-related signalling. P-value < 0.05

Promoter methylation analysis using the UALCAN database revealed that THBS2, CTNNB1, COL4A1, and E2F3 exhibited significantly lower promoter methylation levels in STAD tumor tissues compared to normal controls (Fig. 4 A). To confirm this regulatory relationship, the correlation between promoter methylation and gene expression was examined using GSCA. A significant negative correlation was found for all four hub genes, with Spearman correlation coefficients of − 0.3 for THBS2, − 0.28 for CTNNB1, − 0.13 for COL4A1, and − 0.25 for E2F3 (all FDR < 0.05), indicating that decreased promoter methylation is associated with increased gene expression (Fig. 4B). Further pathway activity analysis via the GSCA database demonstrated that these hub genes were involved in the regulation of key cancer-associated signalling pathways. THBS2, COL4A1, and E2F3 were found to activate epithelial-mesenchymal transition (EMT)-related pathways, while CTNNB1 was strongly associated with the inhibition of hormone receptor-related pathways (Fig. 4 C). Additionally, E2F3 was shown to activate cell cycle-related pathways, reinforcing its role in tumor proliferation (Fig. 4 C).

### Mutational and CNV analysis of hub genes in STAD

**Fig. 5:**
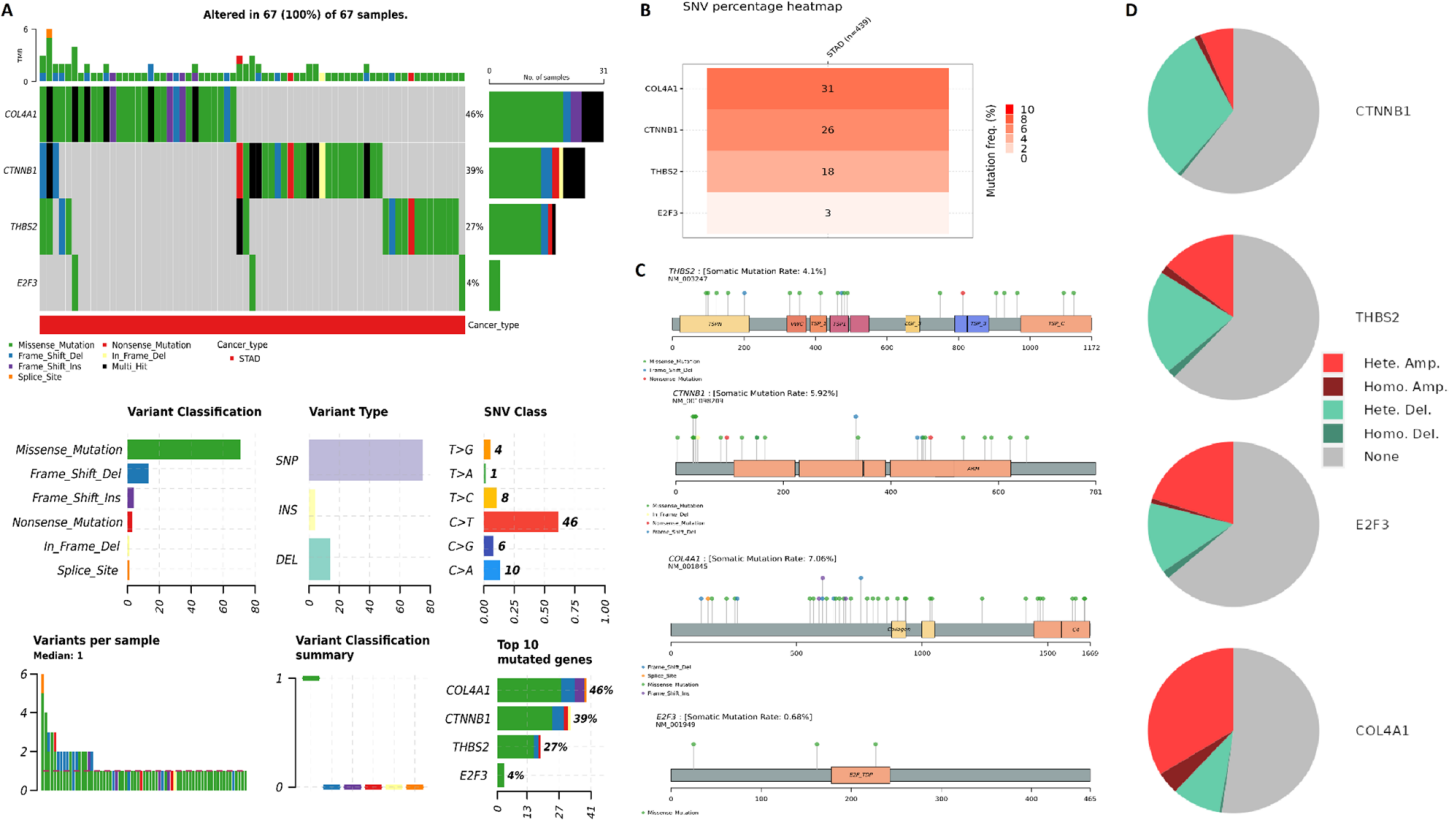
Mutational landscape and copy number variation (CNV) profiling of hub genes in STAD. **A** Summary of variant classifications and types for COL4A1, CTNNB1, THBS2, and E2F3 in STAD samples from the cBioPortal database. **B** Bar plot showing the mutation frequency of each hub gene, with COL4A1 having the highest frequency (46%), followed by CTNNB1 (39%), THBS2 (27%), and E2F3 (4%). **C** Lollipop plots displaying gene-specific mutation positions across protein domains. Somatic mutation rates were 7.06% for COL4A1, 5.92% for CTNNB1, 4.1% for THBS2, and 0.68% for E2F3. **D** Copy number variation (CNV) profiles for each hub gene across STAD samples. P-value < 0.05

Mutation analysis was conducted to investigate the mutational profile of the identified hub genes (COL4A1, CTNNB1, THBS2, and E2F3) in STAD patients using the cBioPortal database. Among the 67 samples analyzed, 100% exhibited genetic alterations, with COL4A1 showing the highest mutation frequency (46%), followed by CTNNB1 (39%), THBS2 (27%), and E2F3 (4%) (Fig. 5A-B). The most common variant classification across the hub genes was missense mutations, followed by frame shift deletions and nonsense mutations. In terms of variant type, single nucleotide polymorphisms (SNPs) were predominant, particularly involving C > T transitions, which constituted the major single nucleotide variant (SNV) class observed (Fig. 5A-B). Gene-specific mutation mapping further revealed that THBS2, CTNNB1, COL4A1, and E2F3 exhibited multiple mutation loci, with CTNNB1 showing a somatic mutation rate of 5.92%, COL4A1 at 7.06%, THBS2 at 4.1%, and E2F3 at 0.68% (Fig. 5 C). These mutations were distributed across the protein domains, including both functional and non-functional regions, indicating potential disruption of protein activity (Fig. 5 C). To complement the mutational analysis, CNV profiling of the hub genes was performed using the cBioPortal database (Fig. 5D). CTNNB1 and E2F3 predominantly exhibited heterozygous deletions, whereas COL4A1 and THBS2 demonstrated a broader spectrum of alterations, including heterozygous and homozygous amplifications in addition to deletions (Fig. 5D). The frequency of CNV alterations varied across genes, with THBS2 and COL4A1 showing a higher prevalence of amplifications (Fig. 5D).

### miRNA–mRNA interaction and diagnostic relevance of key MiRNAs targeting hub genes

**Fig. 6:**
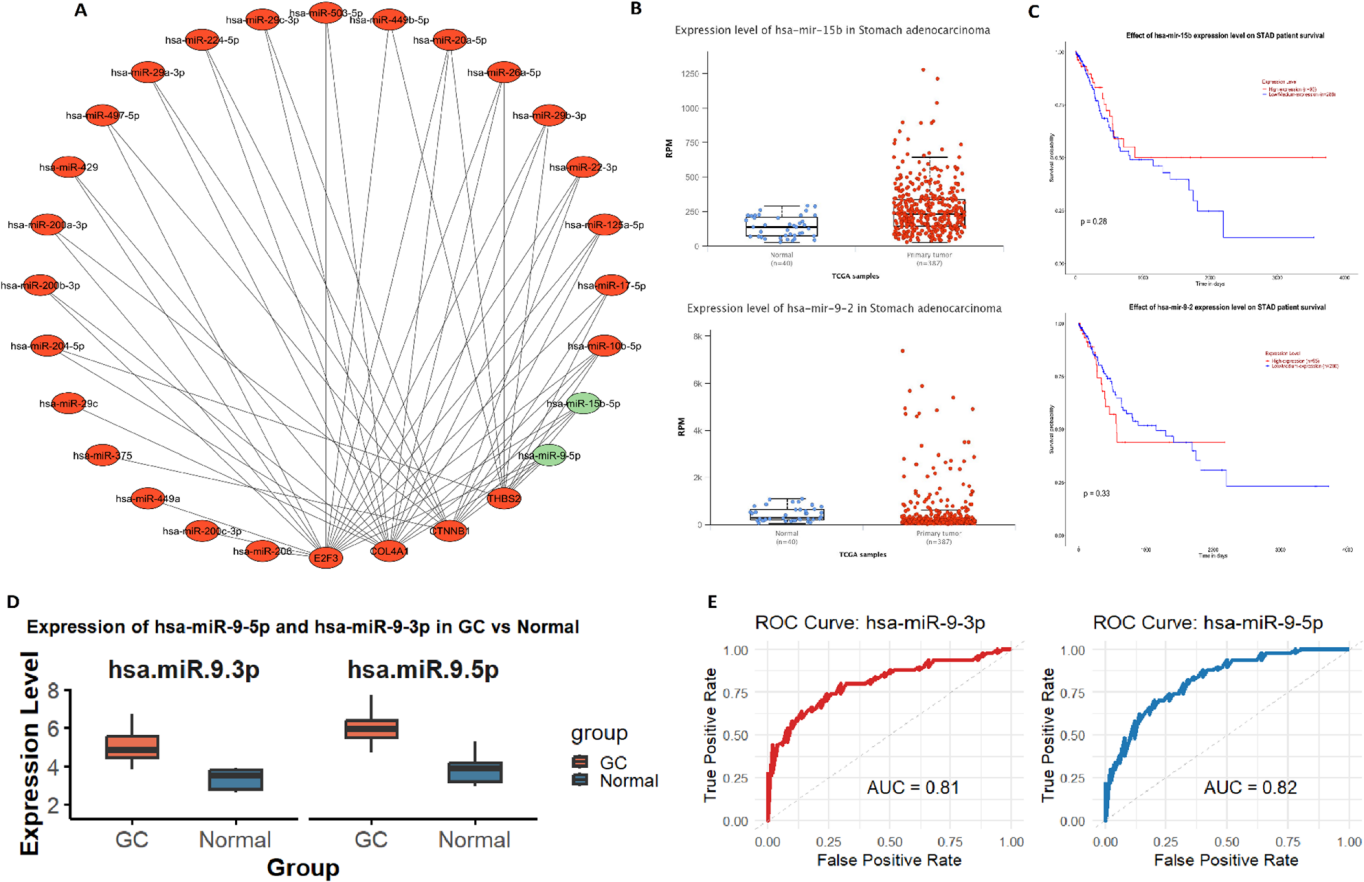
miRNA–mRNA regulatory network and diagnostic value of miRNAs targeting hub genes in STAD. **A** miRNA–mRNA interaction network constructed using the miRNet database. **B** Expression analysis of key miRNAs (hsa-miR-15b and hsa-miR-9-2) in STAD vs. normal tissues using UALCAN database. **C** Kaplan–Meier survival analysis showing no significant association between the expression of hsa-miR-9-3p and hsa-miR-9-5p and overall survival in STAD patients. **D** RT-qPCR validation of hsa-miR-9-3p and hsa-miR-9-5p expression in eight GC cell lines and five normal gastric epithelial cell lines, confirming their upregulation in cancer. **E** ROC analysis demonstrating the diagnostic accuracy of hsa-miR-9-3p (AUC = 0.81) and hsa-miR-9-5p (AUC = 0.82) for distinguishing STAD cells from normal counterparts. P-value < 0.05

To explore post-transcriptional regulation of the identified hub genes (COL4A1, CTNNB1, THBS2, and E2F3), a miRNA–mRNA regulatory network was constructed using the miRNet database. The analysis revealed that hsa-miR-9-3p and hsa-miR-9-5p simultaneously targeted all four hub genes, suggesting a potential coordinated regulatory mechanism at the miRNA level (Fig. 6 A). Expression analysis using the UALCAN database demonstrated that both hsa-miR-15b and hsa-miR-9-2 were significantly upregulated in STAD tissues compared to normal tissues (Fig. 6B). However, Kaplan–Meier survival analysis indicated no statistically significant association between the expression levels of these miRNAs and overall survival in STAD patients (*p* > 0.05) (Fig. 6 C). Subsequent in vitro validation of hsa-miR-9-3p and hsa-miR-9-5p expression in eight gastric cancer cell lines and five normal gastric epithelial cell lines by RT-qPCR confirmed their significant upregulation in gastric cancer (Fig. 6D). To assess their diagnostic potential, ROC analysis was performed. The results showed strong diagnostic value, with AUC values of 0.81 for hsa-miR-9-3p and 0.82 for hsa-miR-9-5p, indicating high sensitivity and specificity in distinguishing gastric cancer from normal samples (Fig. 6E).

### Immune subtype-specific expression, immune landscape, and drug resistance profiling of hub genes in STAD

**Fig. 7:**
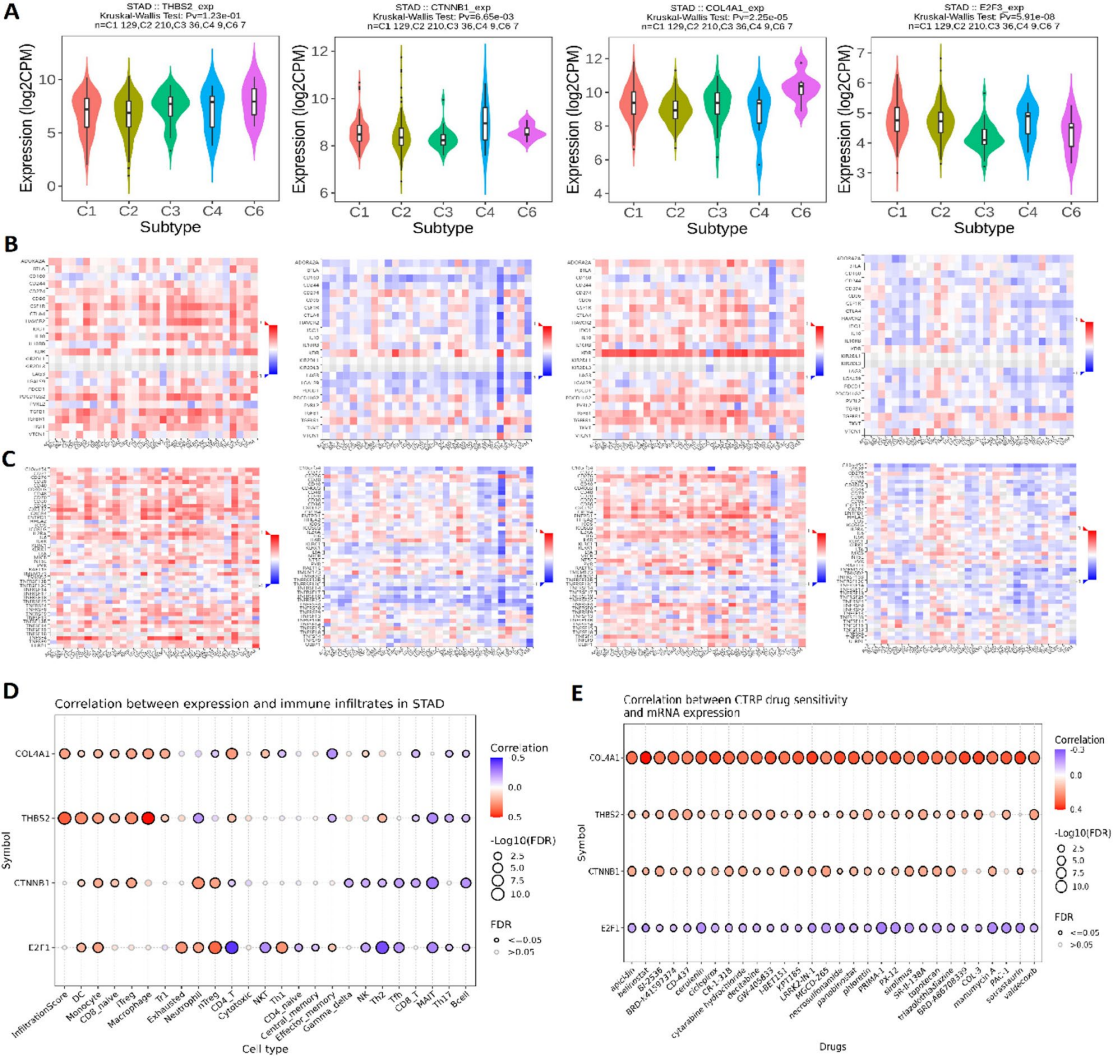
Immune subtype-specific expression, immune modulation, and drug resistance correlations of hub genes in STAD. **A** Expression levels of THBS2, CTNNB1, COL4A1, and E2F3 across STAD immune subtypes. **B** Correlation heatmap showing positive associations (red clusters) between hub genes and immune inhibitory molecules. **C** Correlation heatmap displaying negative associations (blue regions) between hub genes and immune stimulatory molecules. **D** Immune infiltration analysis indicating strong positive correlations of COL4A1 and THBS2 with immunosuppressive cells. **E** Correlation analysis of gene expression with drug resistance profiles from the CTRP database. P-value < 0.05

To evaluate immune context-specific expression, the levels of THBS2, CTNNB1, COL4A1, and E2F3 were analyzed across five immune subtypes (C1, C2, C3, C4, and C6) of STAD using the TISIDB database. All four hub genes demonstrated statistically significant differences in expression among immune subtypes (Fig. 7 A), with CTNNB1 and COL4A1 particularly elevated in the C2 and C6 subtypes, which are generally associated with enhanced immune suppression and tumor aggressiveness (Fig. 7 A). Correlation analysis between hub gene expression and immune inhibitory molecules revealed several red clusters indicating strong positive associations (Fig. 7B). For instance, COL4A1 and THBS2 were positively correlated with inhibitory immune checkpoints such asADORA2A and HAVCR2 (TIM-3) (Fig. 7B). In contrast, correlation analysis with immune stimulatory molecules showed multiple blue regions, denoting significant negative associations (Fig. 7 C). Notably, higher expression of THBS2 and COL4A1 was negatively correlated with molecules like TNFSF13 and TNFRSF25, indicating a potential suppression of immune-activating signals (Fig. 7 C). Further immune infiltration analysis using the GSCA database (Fig. 7 C) revealed gene-specific associations with various immune cell populations. COL4A1 and THBS2 showed strong positive correlations with immunosuppressive cells, such as macrophages and myeloid-derived suppressor cells (MDSCs), reinforcing their potential role in establishing an immune-tolerant tumor microenvironment (Fig. 7D). Finally, drug sensitivity profiling using the CTRP database (Fig. 7E) identified positive correlations (represented by red dots) between the expression of COL4A1, THBS2, and CTNNB1 and resistance to several chemotherapeutic and targeted agents. These correlations suggest that high expression of these genes may serve as predictive markers of poor response to specific drugs, potentially guiding personalized treatment strategies in STAD.

### Gene enrichment analysis

**Fig. 8:**
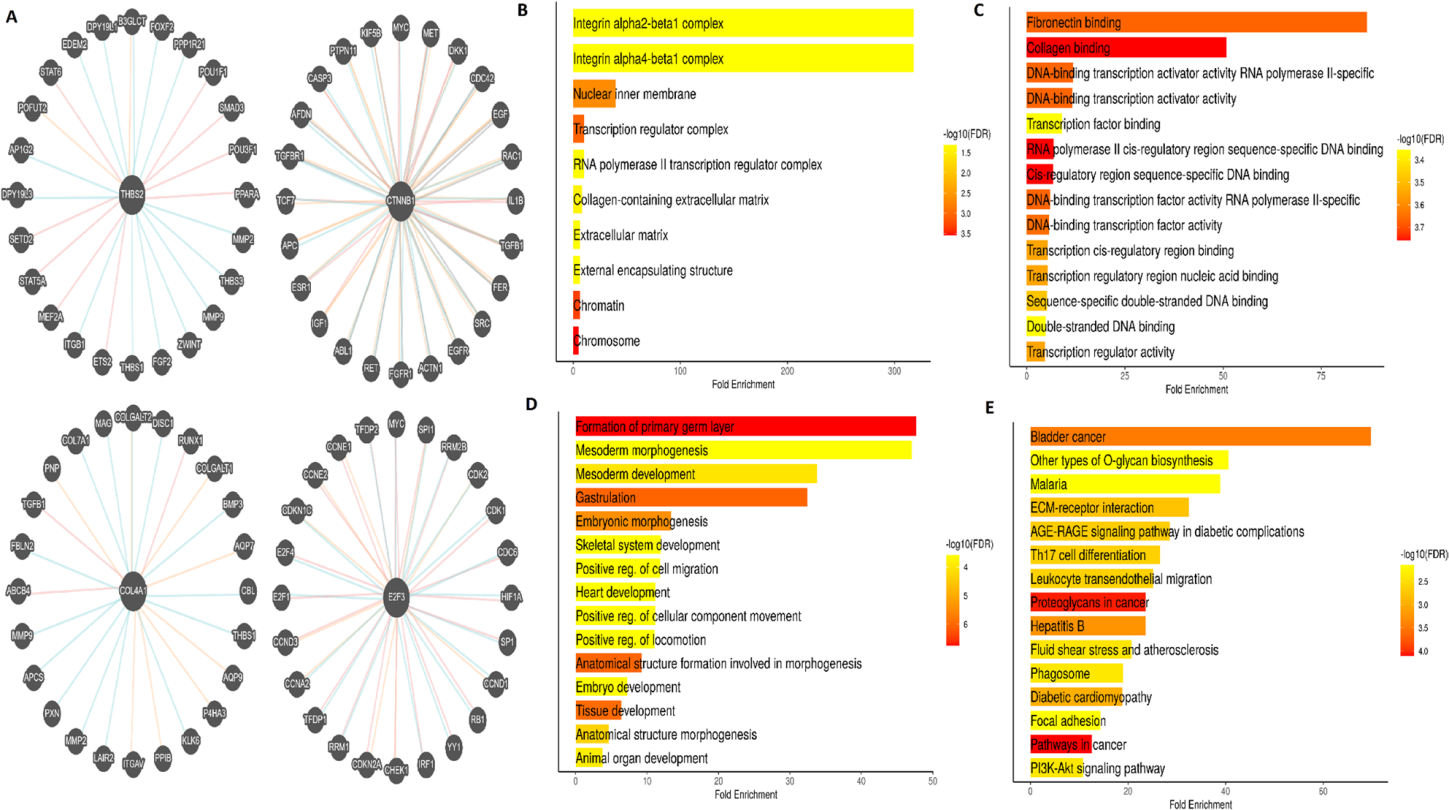
Gene enrichment analysis of hub genes and their interacting partners in STAD. **A** Protein–protein interaction (PPI) network of THBS2, CTNNB1, COL4A1, and E2F3 and their binding partners constructed using the Pathway Commons database. **B** Cellular component (CC) enrichment analysis using DAVID tool, showing significant enrichment in integrin complexes, nuclear inner membrane, chromatin, chromosome, and collagen-containing extracellular matrix. **C** Molecular function (MF) enrichment indicating involvement in fibronectin and collagen binding, transcription activator activity, cis-regulatory region binding, and double-stranded DNA binding. **D** Biological process (BP) enrichment showing association with formation of the primary germ layer, mesoderm morphogenesis, gastrulation, skeletal system development, and positive regulation of cell migration. **E** KEGG pathway analysis highlighting significant enrichment in cancer-related pathways including proteoglycans in cancer, PI3K-Akt signaling, bladder cancer, and immune-related pathways such as AGE-RAGE signaling and Th17 cell differentiation. P-value < 0.05

PPI network analysis via Pathway Commons database identified various interacting partners of THBS2, CTNNB1, COL4A1, and E2F3 (Fig. 8 A). To investigate the functional context of these hub genes and their associated proteins, gene enrichment analysis was conducted using the DAVID tool. Cellular component (CC) enrichment revealed significant overrepresentation in complexes such as integrin alpha2-beta1 and integrin alpha4-beta1, as well as subcellular structures like the nuclear inner membrane, chromatin, chromosome, and collagen-containing extracellular matrix, suggesting involvement of these genes in both intracellular regulation and extracellular matrix interactions (Fig. 8B). Molecular function (MF) enrichment indicated predominant involvement in fibronectin and collagen binding, as well as multiple DNA-binding and transcription-related functions, including transcription activator activity, cis-regulatory region binding, and double-stranded DNA binding. These findings imply a dual role of hub genes in matrix remodeling and transcriptional regulation (Fig. 8 C). Biological process (BP) analysis showed significant enrichment in formation of the primary germ layer, mesoderm morphogenesis, gastrulation, and various aspects of embryonic and tissue development. Other highly enriched terms included skeletal system development, positive regulation of cell migration, and heart development, suggesting a fundamental role for these genes in developmental processes and cell motility (Fig. 8D). Pathway analysis using KEGG identified associations with several cancer-related and signalling pathways. Notably, genes were enriched in “bladder cancer, proteoglycans in cancer, pathways in cancer, and PI3K-Akt signaling pathway.” Additional enrichment was seen in “immune-related and metabolic pathways such as AGE-RAGE signalling in diabetic complications, Th17 cell differentiation, and fluid shear stress and atherosclerosis,” pointing to a diverse involvement in oncogenic and inflammatory signalling cascades (Fig. 8E).

### H. pylori induce COL4A1/CTNNB1 activation and promotes malignant, while gene Silencing reverses these effects

**Fig. 9:**
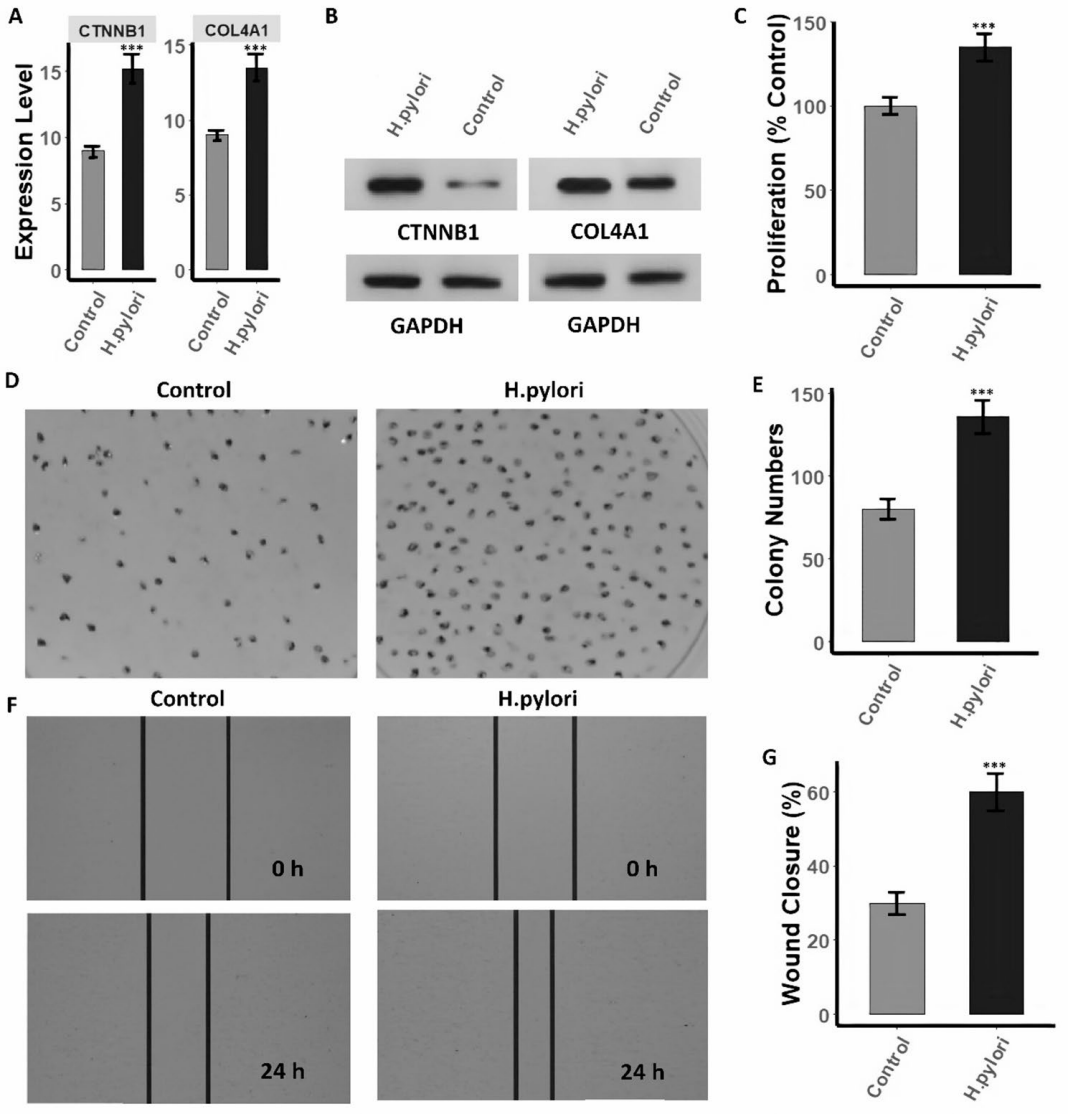
H. pylori infection upregulates COL4A1 and CTNNB1 and enhances proliferation, clonogenicity, and migration in AGS cells. **A** RT-qPCR analysis showing significant induction of COL4A1 and CTNNB1 mRNA levels in H. pylori-infected AGS cells compared with uninfected controls. **B** Western blot analysis confirming increased COL4A1 and CTNNB1 protein abundance following infection. **C** CCK-8 assay demonstrating higher proliferation (percent of control) in infected cells at 24 hours. **D**-**E** Colony formation assay showing increased colony numbers. **F**-**G** Wound-healing assay indicating accelerated migration in infected cells. P***-value < 0.001

**Fig. 10:**
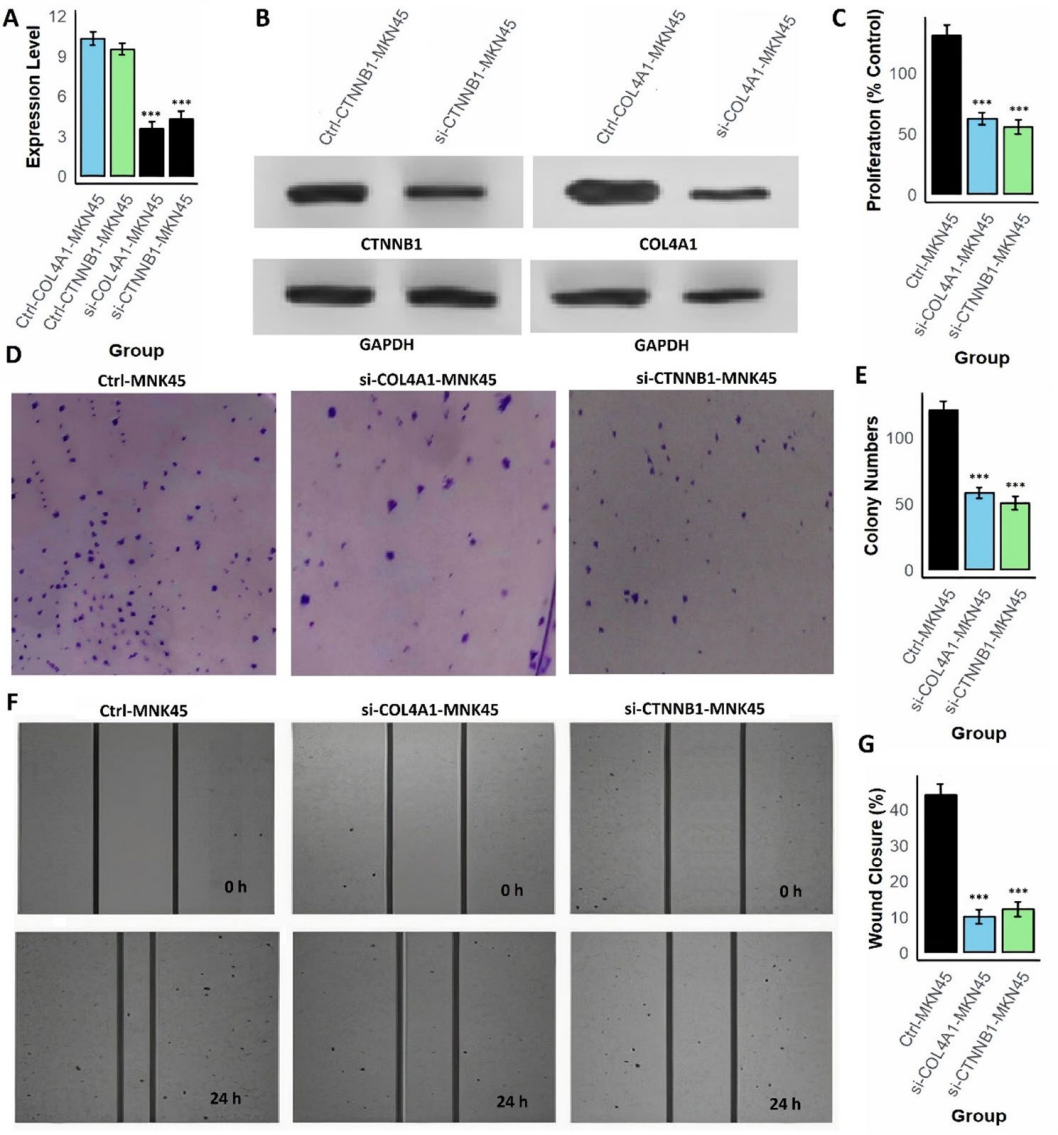
Functional characterization of COL4A1 and CTNNB1 in MNK45 cells. **A** RT-qPCR analysis confirming efficient knockdown of COL4A1 and CTNNB1 mRNA expression in MNK45 cells following siRNA transfection. **B** Western blot validation showing reduced protein expression of COL4A1 and CTNNB1 in MNK45 cells post-silencing. **C** Cell proliferation assay demonstrating a significant decrease in MNK45 cell proliferation upon silencing of COL4A1 or CTNNB1. **D**-**E** Colony formation assay showing impaired clonogenic potential in MNK45 cells following knockdown of COL4A1 or CTNNB1. **F**-**G** Wound healing assay revealing reduced migratory capacity of MNK45 cells after gene silencing, as evidenced by lower wound closure percentages at 24 hours. P***-value < 0.001

**Fig. 11:**
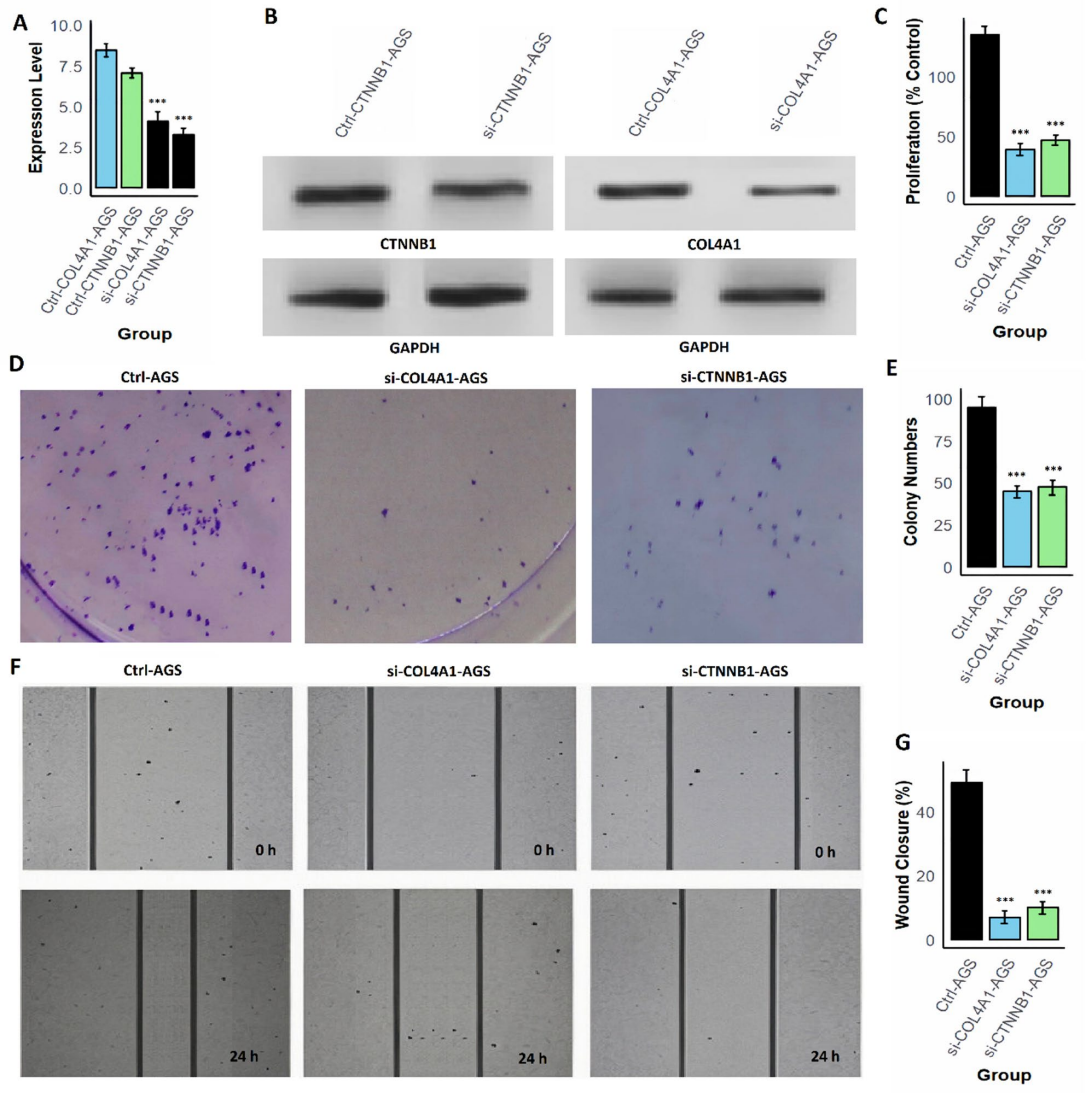
Functional characterization of COL4A1 and CTNNB1 in AGS cells. **A** RT-qPCR analysis confirming successful silencing of COL4A1 and CTNNB1 mRNA expression in AGS cells. **B** Western blot analysis showing downregulation of COL4A1 and CTNNB1 protein levels in AGS cells following siRNA treatment. **C** Cell proliferation assay indicating a significant reduction in AGS cell proliferation upon knockdown of COL4A1 or CTNNB1. **D**-**E** Colony formation assay demonstrating marked suppression of colony-forming ability in AGS cells after silencing of either gene. **F**-**G** Wound healing assay showing impaired cell migration in AGS cells following knockdown of COL4A1 or CTNNB1, with decreased wound closure observed after 24 hours. P***-value < 0.001

To investigate whether H. pylori infection directly enhances malignant phenotypes through COL4A1 and CTNNB1, AGS cells were first infected with live H. pylori. RT-qPCR and Western blot analysis revealed a strong induction of both gene expression in infected AGS cells compared with uninfected controls (Fig. 9A–B and supplementary data Fig. 1). Furthermore, CCK-8 assays showed a 35% increase in proliferation at 24 h (Fig. 9 C), and colony formation assays demonstrated nearly 1.7-fold more colonies in infected cells than controls (Fig. 9D–E). Infection also accelerated cell migration, as wound-healing assays revealed approximately a two-fold increase in wound closure within 24 h (Fig. 9F-G). These results indicate that H. pylori promote aggressive phenotypes in AGS cells at least partly through upregulating COL4A1 and CTNNB1. To verify the functional importance of these two hub genes, loss-of-function experiments were subsequently performed in MNK45 and AGS cells using siRNAs targeting COL4A1 and CTNNB1. Knockdown efficiency was confirmed by RT-qPCR and Western blotting, which showed substantial reductions in both mRNA and protein levels (Fig. 10, 11 A–B and supplementary data Fig. 1). Suppression of either COL4A1 or CTNNB1 resulted in significant inhibition of cell proliferation, with CCK-8 assays showing a marked decrease relative to control groups (Fig. 10, 11 C). Clonogenicity was similarly impaired, as colony formation assays demonstrated a pronounced reduction in colony number and size following gene silencing (Figs. 10 and 11D–E). Furthermore, cell migration was strongly attenuated, with wound closure rates dramatically lower in knockdown cells at 24 h compared to controls (Fig. 10, 11 F–G).

## Discussion

In this study, we employed an integrative bioinformatics and experimental approach to identify and functionally characterize H. pylori-associated hub genes in stomach adenocarcinoma (STAD) [57, 58]. Through transcriptomic analysis of two independent datasets, we identified four hub genes, including THBS2, CTNNB1, COL4A1, and E2F3 which were consistently dysregulated in H. pylori-positive STAD samples. Our observed upregulation of CTNNB1 and COL4A1 is consistent with earlier studies reporting elevated expression of these genes in gastric cancer tissues, particularly in the presence of H. pylori infection [59, 60]. Similarly, previous transcriptomic analyses have documented increased THBS2 and E2F3 expression in advanced gastric tumors [61, 62], which aligns with the significant overexpression detected across our datasets and validated in STAD cell lines. These findings are also in line with previous systems biology efforts that have implicated extracellular matrix (ECM) remodeling, Wnt signaling, and cell cycle dysregulation in gastric tumorigenesis [63–65].

THBS2, a matricellular glycoprotein, has been previously implicated in promoting tumor angiogenesis and metastasis [66, 67]. In agreement with these roles, we found that THBS2 was overexpressed in STAD tissues and cell lines and showed strong prognostic value with the highest AUC among the identified genes. Similarly, CTNNB1, which encodes β-catenin—a key effector of canonical Wnt signaling—was also upregulated and associated with poor survival [68, 69]. This finding corroborates earlier studies reporting Wnt/β-catenin pathway activation as a downstream consequence of CagA-positive H. pylori infection, which stabilizes β-catenin and drives gastric epithelial transformation [70, 71].

COL4A1, a component of the basement membrane, was shown to be progressively upregulated across tumor stages, suggesting its involvement in disease advancement. Previous work has shown that COL4A1 contributes to ECM remodeling and invasive behaviour in multiple cancer types [72, 73]. Likewise, E2F3, a known regulator of cell cycle progression, was associated with enhanced proliferation and EMT activation in our pathway analyses [74, 75]. Prior studies have indicated that E2F3 overexpression promotes gastric tumor cell growth and correlates with poor patient outcomes [76, 77].

Our comprehensive gene enrichment analyses further support the biological significance of these hub genes. Consistent with prior work [78–80], we found enrichment in cellular components related to integrin and ECM complexes, molecular functions involving DNA-binding and transcription regulation, and biological processes such as germ layer formation, morphogenesis, and cell migration. The KEGG analysis revealed involvement in multiple cancer-associated pathways, including PI3K-Akt, ECM–receptor interaction, and proteoglycans in cancer—many of which have previously been shown to be activated in the context of H. pylori-driven carcinogenesis [81].

Importantly, we performed in vitro validation to experimentally confirm the roles of COL4A1 and CTNNB1. Gene silencing via siRNA significantly suppressed proliferation, colony formation, and wound healing ability in both MNK45 and AGS cells. These findings are consistent with previous functional studies of CTNNB1, where its knockdown reduced tumor cell viability and migratory capacity in gastric and colorectal cancer models [82, 83]. Our findings for COL4A1 are also supported by recent reports demonstrating that its depletion impairs proliferation and invasion in hepatocellular carcinoma and glioma cells [84, 85], although its specific role in STAD had not been well characterized prior to this study. This emphasizes our contribution in extending the understanding of COL4A1 as a critical regulator in gastric cancer progression, particularly in the context of H. pylori-associated malignancy.

We also observed that these hub genes are epigenetically regulated via promoter hypomethylation—a mechanism that has been reported in several oncogenes activated in STAD [86, 87]. For CTNNB1 and COL4A1, our findings are consistent with earlier studies that have noted reduced methylation accompanying their elevated expression in STAD [88, 89]. In contrast, promoter hypomethylation of THBS2 and E2F3 has been less frequently documented, suggesting that the patterns identified in our analysis may represent comparatively novel methylation related changes associated with H. pylori driven STAD. Furthermore, mutation and CNV analyses revealed frequent genomic alterations in these genes, especially COL4A1 and CTNNB1, reinforcing their potential as drivers of tumor evolution. Our observation of recurrent CTNNB1 alterations aligns with the earlier findings, confirming its well established role as a genetically unstable and functionally significant gene in STAD [90]. In contrast, the high frequency of COL4A1 alterations observed in our analysis appears less widely reported in previous STAD studies, indicating that COL4A1 may represent a comparatively under recognized mutational contributor to gastric tumor progression. This combination of expected and newly highlighted genomic patterns strengthens the relevance of our results while expanding current understanding of mutation and CNV involvement in H. pylori associated STAD. Prior studies have described CTNNB1 mutations in STAD and their association with enhanced Wnt signaling and immune evasion [91, 92].

Interestingly, immune correlation analyses revealed that COL4A1 and THBS2 were positively correlated with immunosuppressive checkpoints such as HAVCR2 and ADORA2A and showed enrichment in immunosuppressive cell populations including MDSCs and macrophages. These results align with recent findings linking tumor ECM components to immune evasion through physical and biochemical modulation of the tumor microenvironment [93, 94]. Previous studies in stomach adenocarcinoma have similarly shown that dense ECM deposition can restrict T cell infiltration and interfere with the effectiveness of checkpoint blockade therapies, particularly those targeting PD1 and CTLA4 [95]. Moreover, several immunotherapies based clinical analyses have reported that stromal rich gastric tumors exhibit reduced responses to immune checkpoint inhibitors [96], which supports our observation that COL4A1 and THBS2 correlate with markers of an immune suppressive microenvironment. The association of these genes with macrophages and myeloid derived suppressor cells further resonates with earlier work indicating that ECM remodeling promotes recruitment of suppressive myeloid cells, thereby weakening antitumor immunity [96].

Our analysis identified hsa-miR-9-3p and hsa-miR-9-5p as shared regulators of COL4A1, CTNNB1, THBS2, and E2F3, a pattern that aligns partially with earlier studies reporting dysregulation of the miR-9 family in gastric cancer [97]. Previous reports have shown that miR-9 members are frequently upregulated in gastric tumors and can promote proliferation, migration, and inflammatory signaling [98, 99]. The significant overexpression of miR-9-3p and miR-9-5p in our STAD cell lines is consistent with these findings, reinforcing the idea that miR-9 family members participate broadly in gastric tumorigenesis. However, the simultaneous targeting of all four of our identified hub genes has not been described previously and suggests a more extensive regulatory influence than reported in earlier single-target studies. Interestingly, unlike some prior studies that linked elevated miR-9 expression to poor patient prognosis [100, 101], our Kaplan–Meier analysis showed no association with overall survival, indicating a potential divergence in clinical relevance across cohorts. This suggests that miR-9-3p and miR-9-5p may function predominantly as diagnostic markers rather than prognostic indicators in STAD.

The drug resistance profiling further indicated that high expression of COL4A1, THBS2, and CTNNB1 was associated with reduced sensitivity to a range of chemotherapeutic agents, suggesting that these genes may contribute to an intrinsic or acquired drug-resistant phenotype in STAD. These findings align with prior studies that ECM components in forming a protective barrier that limits drug penetration, activates integrin-mediated survival signaling, and promotes EMT—all of which are known contributors to therapy resistance. Specifically, COL4A1 has been linked to enhanced matrix stiffness and pro-survival cues that counteract apoptosis induced by cytotoxic drugs [102, 103]. Similarly, CTNNB1, a central component of the Wnt signaling pathway, has been shown to drive resistance through transcriptional activation of genes involved in drug efflux, stemness, and DNA repair [104]. THBS2, an ECM glycoprotein, has also been implicated in modulating tumor angiogenesis and immune evasion, which may further compromise drug efficacy [105]. Therefore, the observed positive correlations between these genes and resistance to agents such as 5-fluorouracil, oxaliplatin, and irinotecan not only highlight their prognostic relevance but also emphasize their potential as therapeutic targets for overcoming chemoresistance in STAD. Overall, these observations are consistent with reports from STAD studies where ECM enriched tumors have been found to exhibit poor responses to standard chemotherapy regimens such as fluoropyrimidines and platinum-based therapies [106, 107]. Moreover, recent resistance profiling in gastric cancer has suggested that Wnt beta catenin activation and ECM remodeling cooperate to generate a drug tolerant state [108], which aligns with our findings for CTNNB1 and COL4A1. Incorporating these earlier insights underscores the broader relevance of our resistance related results and strengthens the rationale for considering these genes as potential targets for overcoming therapeutic failure in stomach adenocarcinoma.

Finally, as our understanding of tumor biology continues to expand, cancer treatment is moving steadily toward more individualized and mechanism-driven approaches. Recent discussions in the literature emphasize that future therapies will increasingly rely on strategies that modulate the tumor immune microenvironment, selectively interrupt aberrant signaling pathways, and integrate molecular profiling to guide patient specific interventions [109]. These emerging directions are particularly relevant for H. pylori–associated gastric cancer, where chronic inflammation, stromal remodeling, and pathway dysregulation shape both disease progression and treatment response. In this context, our identification of CTNNB1 and COL4A1 as central regulators provides a strong foundation for developing targeted approaches, since both genes participate in pathways that are highly actionable in modern precision oncology. Therapeutic strategies aimed at controlling aberrant Wnt beta catenin activity or modifying extracellular matrix dynamics could be combined with immune-directed therapies to counteract the immunosuppressive environment characteristic of H. pylori–driven tumors. These possibilities point toward a future in which molecularly informed treatment plans, centered on targets such as CTNNB1 and COL4A1, may offer improved outcomes for patients with H. pylori–related gastric cancer.

Despite its strengths, the study has several limitations. In vivo validation using animal models was not conducted, which limits the physiological relevance of the functional assays. Furthermore, only two of the four identified hub genes were functionally validated, leaving the biological roles of THBS2 and E2F3 unexplored. While miR-9-3p and miR-9-5p were identified as common regulators of all four hub genes and shown to be upregulated in STAD, their direct interaction and functional impact were not validated using luciferase reporter assays or miRNA rescue experiments. In addition, no validation was performed in primary patient biopsy samples, which would have strengthened the clinical applicability of the findings. Furthermore, the use of bulk RNA-seq data restricts insight into tumor heterogeneity and cell-type-specific expression, which could be better addressed using single-cell transcriptomic analysis in future studies. Lastly, beyond the analyses performed here, additional bioinformatic frameworks could further strengthen the understanding of these targets. Recent cancer research published in 2023 has demonstrated how integrating tumor microenvironment quantitative scoring and immune context modelling can reveal how specific genes interact with stromal components, immune infiltrates, and prognostic molecular patterns [110]. Applying similar tumor microenvironment oriented analytical strategies to THBS2, CTNNB1, COL4A1, and E2F3 may offer deeper insight into their roles in shaping immune suppression, stromal remodeling, and treatment response in STAD.

## Conclusion

In summary, this study comprehensively identified and validated COL4A1 and CTNNB1 as critical hub genes involved in H. pylori-associated STAD. Through integrative transcriptomic analysis of two independent H. pylori-related datasets, we identified commonly dysregulated genes, which were then subjected to PPI network construction and hub gene prioritization. Subsequent gene enrichment analyses, expression profiling across molecular subtypes, clinical stages, and survival analyses revealed the oncogenic and prognostic relevance of these genes. Protein-level validation through immunohistochemistry further corroborated their elevated expression in tumor tissues. Importantly, epigenetic and genomic analyses showed that promoter hypomethylation and frequent copy number alterations likely underlie their aberrant overexpression in STAD. Functional validation using siRNA-mediated knockdown in MNK45 and AGS cell lines demonstrated that COL4A1 and CTNNB1 play essential roles in maintaining tumor cell proliferation, clonogenic survival, and migratory potential, confirming their functional importance in gastric cancer progression. Furthermore, immune landscape analyses indicated that these genes are associated with immunosuppressive cell infiltration and negative regulation of immune-stimulatory molecules, suggesting a role in immune evasion. Drug sensitivity profiling revealed positive correlations between their expression and resistance to multiple chemotherapeutic agents, highlighting their involvement in ECM-mediated therapy resistance mechanisms. Collectively, our findings offer novel mechanistic insights into how H. pylori infection drives STAD progression and propose COL4A1 and CTNNB1 as promising diagnostic biomarkers and therapeutic targets. Future studies should aim to validate these findings in animal models and clinical cohorts and investigate the potential of targeting these genes to enhance treatment efficacy in STAD patients.

## Supplementary Information

Supplementary Material 1

## Data Availability

Any type of the data will be provided by the corresponding author.
