# Supplementary material for: Integrative analysis of Helicobacter pylori-driven stomach adenocarcinoma reveals epigenetic deregulation, immune evasion, and therapeutic resistance
Source: Hereditas. 2025 Nov 25;163:1. doi: 10.1186/s41065-025-00616-z (PMC12764169; doi:10.1186/s41065-025-00616-z)
Supplement: Supplementary file 1 — Supplementary Material 1 [file 41065_2025_616_MOESM1_ESM.pdf]

For Figure 9

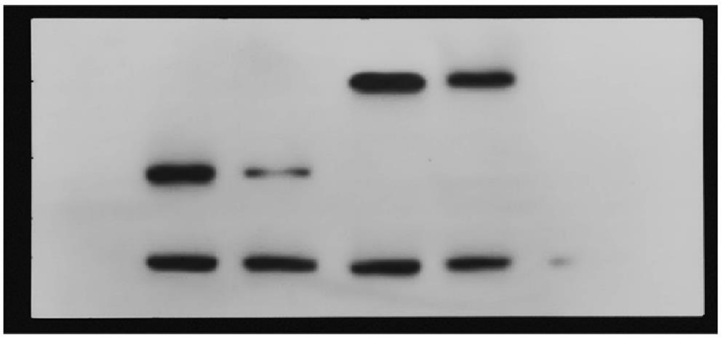

For Figure 10 and 11

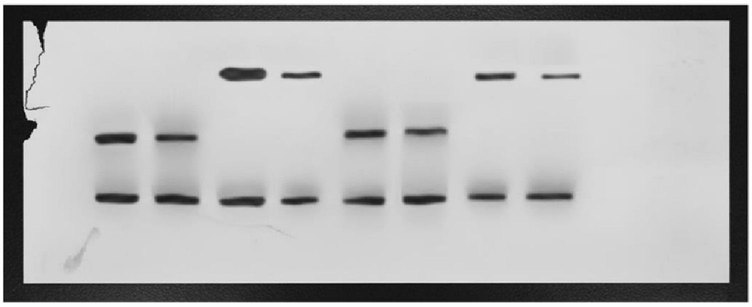

Supplementary data Figure 1: Uncut Western blot bands of COL4A1, CTNNB1, and GAPDH.
